# Elastic pneumatic tourniquet cuff can reduce postoperative thigh pain after total knee arthroplasty: a prospective randomized trial

**DOI:** 10.1186/s12891-020-03579-6

**Published:** 2020-08-21

**Authors:** Jae-Young Park, Sung Eun Kim, Myung Chul Lee, Hyuk-Soo Han

**Affiliations:** Department of Orthopedic Surgery, Seoul National University College of Medicine, 101 Daehak-ro, Jongno-gu, Seoul, 03080 Republic of Korea

**Keywords:** Total knee arthroplasty, Automatic pneumatic tourniquet, Elastic cuff

## Abstract

**Background:**

Tourniquet use is associated with complications such as thigh pain, skin problems, and deep vein thrombosis (DVT). This prospective study aimed to evaluate the efficacy and safety of the pneumatic tourniquet system using an elastic cuff and limb occlusion pressure (LOP) in total knee arthroplasty (TKA). The hypothesis of this study was that an elastic cuff tourniquet would result in less postoperative thigh pain after TKA.

**Methods:**

This prospective randomized controlled trial involved a total of 98 patients who underwent primary TKA. They were randomized into two groups: tourniquet system using an elastic cuff and LOP group (Group E) and tourniquet system using a conventional-cuff and LOP group (Group C). Outcomes including postoperative thigh pain assessed using a visual analog scale (VAS), serum muscle enzymes, recommended tourniquet pressure (RTP), bloodlessness of surgical field, surgical time, incidence of DVT, and the frequency of rescue analgesic use after surgery, were compared between groups.

**Results:**

Patients in Group E experienced significantly less thigh pain compared to those in Group C on postoperative day 4 (*P* = 0.01) and day 7 (*P* = 0.04). The difference between RTP and systolic blood pressure was significantly lower in Group E (*P* = 0.045). One case of thigh DVT was found in Group E, while no such cases were found in Group C. One and two cases of poor bloodless surgical fields were observed in Group E and Group C, respectively. There was no significant difference in surgical time, levels of serum muscle enzymes, and the frequency of rescue analgesic use between the two groups.

**Conclusions:**

The pneumatic tourniquet system using an elastic cuff and LOP reduced early postoperative thigh pain more effectively than did the tourniquet system using a conventional cuff and LOP.

**Trial registration:**

#KCT0003149. Registered August 17, 2018 - Retrospectively registered.

## Background

In many orthopedic surgeries, the pneumatic tourniquet is efficiently used to achieve a bloodless surgical field. It decreases total blood loss during surgery, facilitates cementation, and decreases operative time [1–3], especially in total knee arthroplasties (TKAs). The conventional tourniquet pressure in TKAs is approximately 300–350 mmHg. High pressure may lead to postoperative complications such as ischemic pain, swelling, skin abrasions, blisters, rhabdomyolysis, deep vein thrombosis (DVT), wound healing disorders, and nerve palsy [4–8].

Limb occlusion pressure (LOP) is the tourniquet pressure needed to occlude the blood flow. LOP is influenced by many factors including age, systolic blood pressure, materials or width of the cuff, and limb circumference [9, 10]. Conventional cuffs are not flexible and may create folds on the inner side. These folds can cause postoperative pain, pinching of the skin, and leakage of blood flow in the surgical field. These folds also cause the pressure of the cuff to be unevenly transmitted to the thigh. Thus, choosing a cuff made of an appropriate material, as well as adjusting the tourniquet pressure is necessary to reduce postoperative complications and to maximize the effectiveness of the tourniquet system. Theoretically, elastic cuffs may distribute the tourniquet pressure to the thigh evenly [11]. Thus, the use of elastic cuffs may be advantageous for decreasing tourniquet pressure, and to reduce thigh pain and incidence of DVT [11]. However, studies comparing the raw material of the cuff to reduce the complications associated with tourniquets have rarely been conducted.

In a previous pilot study, clinical outcomes of TKAs with elastic cuffs and conventional cuffs were compared. There was no significant difference in the outcomes between the two groups. However, the number of subjects was small and outcome analysis was performed for only 7 days postoperatively [11]. In this study, we aimed to evaluate the efficacy and safety of a tourniquet system using elastic cuffs and LOP in TKAs. We hypothesized that compared to a conventional cuff tourniquet, an elastic cuff tourniquet would result in lower thigh pain after TKA, as well as in a lower incidence of complications such as DVT and skin problems.

## Methods

This study was a prospective randomized controlled trial comparing the clinical outcomes of TKAs using an elastic cuff tourniquet or a conventional cuff tourniquet at a single center. The current trial was registered at cris.nih.go.kr (#KCT0003149). This study was approved by the institutional review board.

Eligible participants were defined as follows: all patients with primary osteoarthritis aged between 50 and 80 years, who were scheduled for primary TKA. Exclusion criteria were as follows: a history of anticoagulation therapy, bleeding disorders, DVT, psychiatric illness, active malignancy, chronic alcoholism, or previous surgery of the extremities.

Patients were randomly assigned to receive TKA with an elastic tourniquet cuff (Group E) or with a conventional tourniquet cuff (Group C). For Group E, a pneumatic tourniquet system using an elastic cuff and LOP (DTS-3000, DSMAREF, Gunpo, South Korea) was employed. LOP was measured by placing a pulse oximetry sensor on a toe digit of the foot corresponding to the leg being operated on. The cuff automatically inflates to a pressure high enough that the patient’s pulse signal is not detected, and then deflates while the patient’s pulse signal is detected; the sensor parameters are adjusted automatically. The recommended tourniquet pressure (RTP) was automatically set based on the automatically measured LOP plus a safety margin using the modified McEwen’s guideline, for both tourniquet systems [12]. The inner membrane of the elastic cuff is made of silicone rubber to prevent any pinching of the skin and excess pressure of the tourniquet. For Group C, a pneumatic tourniquet system using conventional cuffs and LOP (ATS-4000, Zimmer, Warsaw, USA) was used. Conventional cuffs are made of a polyurethane elastomer. Primary TKA was performed using the same technique in both groups, as described in a previous study [13].

Patients were randomized using a random number generator with permuted blocks of 10. Allocations were kept in sequentially numbered and sealed envelopes, which were opened prior to the surgery, after the patient had given written consent. The research coordinator and the patient were blinded to treatment allocation.

The primary outcome was postoperative thigh pain assessed postoperatively at day 4 using a visual analog scale (VAS) ranging from 0 to 10.

Secondary outcomes were postoperative thigh pain assessed postoperatively at day 1,2, and 7 and the levels of creatine phosphokinase (CPK) and lactate dehydrogenase (LDH) on postoperative days 1, 2, 4, and 7.

The RTP of the patients was recorded, and the systolic blood pressure (SBP) at the time of the measurement of LOP was also recorded. During surgery, surgeons evaluated the bloodlessness of the surgical field, based on the findings of a previous study [14]. The surgical field was evaluated as poor, fair, good, or excellent. Surgical time was recorded. The frequency of rescue analgesic use was measured during hospital stay. Complications such as skin problems of the thigh and the surgical wound were inspected. Duplex ultrasonography for DVT evaluation was performed preoperatively and on postoperative day 7. Occurrences of DVT above and below the knee were recorded. If DVT was diagnosed, a follow-up study was performed at 3 months and 6 months postoperatively.

### Sample size and statistical analysis

Sample size was estimated to detect one-point differences in the postoperative thigh pain VAS scores. The VAS data were assessed by an independent t-test to define clinically meaningful differences. Based on the data from a previous study [11], at least 48 knees in each group were required to detect this difference. The type 1 error was 0.05, and the power was 0.8.

Descriptive data were reported as means and standard deviations (SDs) for continuous values. Continuous variables between the two groups were compared using the Student’s t-test and differences in other categorical variables between the two groups were analyzed with chi-square tests and Fisher’s exact tests. Differences in postoperative VAS scores of thigh pain during the postoperative follow-up period in both groups were detected using one-way analysis of variance (ANOVA). Postoperative VAS scores of thigh pain of the two groups were additionally compared using the Student’s t-test. A *P*-value < 0.05 was considered significant. All statistical analyses were performed with the SPSS version 22.0 software (SPSS Corp., Chicago, USA).

## Results

**Fig. 1:**
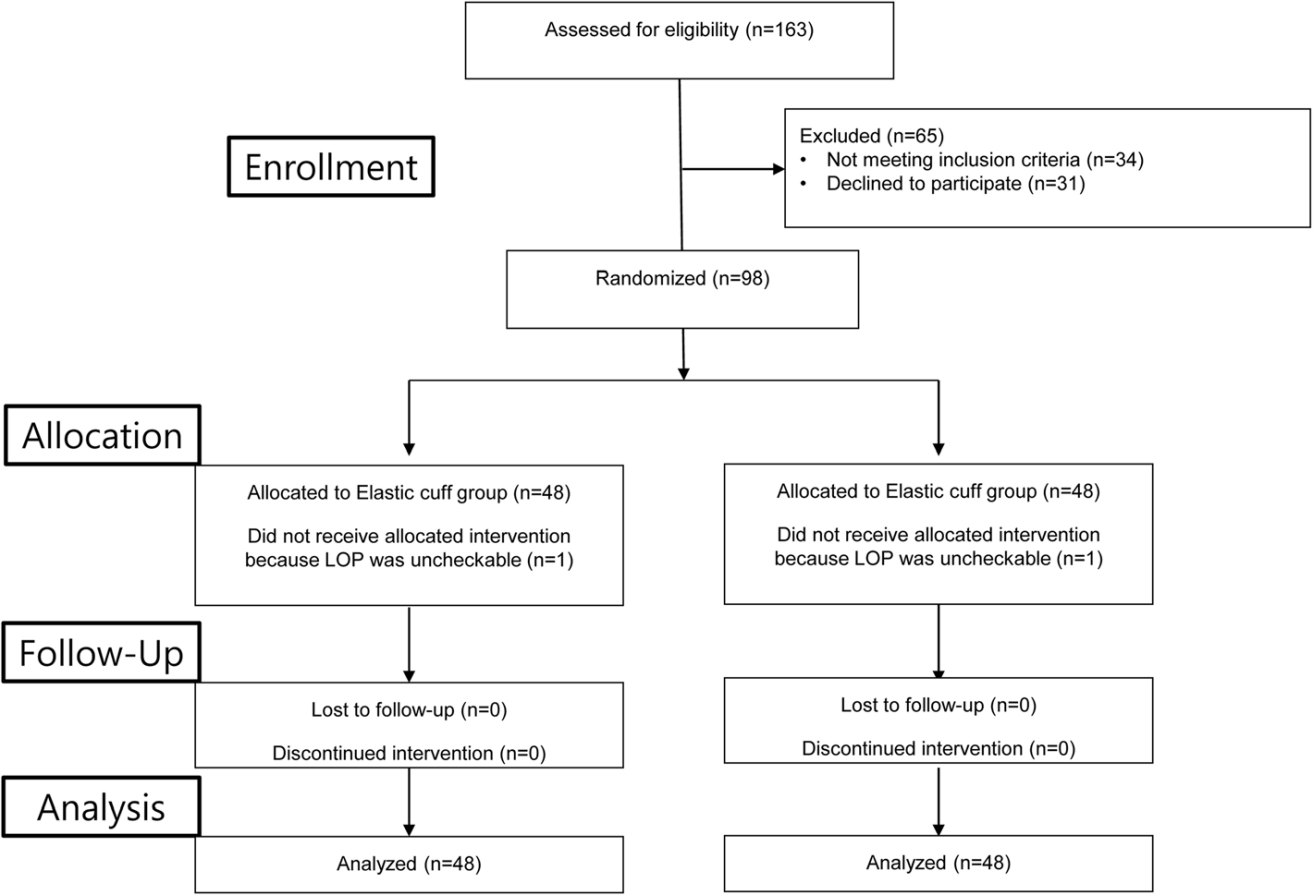
Summary of enrollment. LOP = limb occlusion pressure

Between August 2016 and July 2017, a total of 163 patients were screened for eligibility; 98 patients met the inclusion criteria, and were randomized into the two study groups. In total, 96 patients completed the trial (Fig. 1). There were no significant differences in demographic data between the two groups (Table 1).

**Table 1:** Baseline characteristics of patients

	Group E (*n* = 48)	Group C (*n* = 48)	*P*-value
Male/female	6 / 42	4 / 44	n.s
Age (year)	69.7 ± 4.5	71.9 ± 4.9	n.s
BMI (kg/m^2^)	27.0 ± 3.6	26.2 ± 4.9	n.s
Surgical time (min)	51.4 ± 13.0	50.0 ± 12.3	n.s

The data are expressed as the mean ± standard deviations

*Group E* elastic cuff group, *Group C* conventional cuff group, *BMI* body mass index, *n.s* not significant

### Thigh pain VAS scores

A one-way repeated measure ANOVA was conducted to compare the effect of an elastic cuff tourniquet and that of a conventional cuff tourniquet on VAS scores for thigh pain on different postoperative days. Significant between-group differences were observed in VAS scores for thigh pain (*P* < 0.0001).

**Fig. 2:**
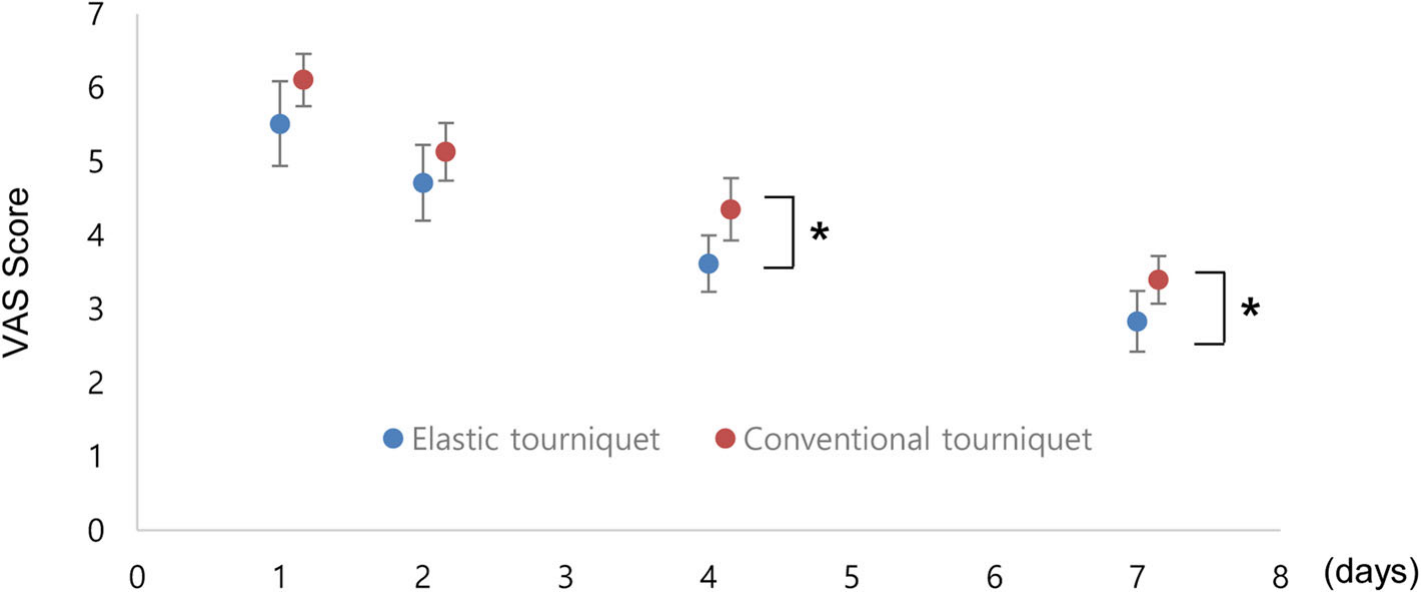
The mean thigh pain visual analog scale was significantly lower in the elastic cuff group on postoperative days 4 and 7. The bars indicate the 95% confidence interval of the mean value. The asterisks indicate values that were significantly different between the two groups (*P* < 0.05)

Patients in Group E experienced significantly lower thigh pain (as per the VAS) compared to those in Group C, on postoperative days 4 and 7 (Fig. 2). On day 4, the VAS score for thigh pain was 3.6 (±1.3) for Group E and 4.3 (±1.4) for Group C (*P* = 0.01). On day 7, the VAS score for thigh pain was 2.8 (±1.4) for Group E and 3.3 (±1.1) for Group C (*P* = 0.04).

### Hemoglobin, hematocrit, and muscle enzymes

Both groups showed significant decreases in hemoglobin and hematocrit values postoperatively (*P* < 0.001) The postoperative levels of hemoglobin or hematocrit were not different between the two groups. (*P*-value: not significant (n.s.)) The level of CPK, a marker of muscular injury, was not different between the two groups. (*P*-value: n.s.) Both groups showed peak elevation of CPK on postoperative day 2. The level of LDH, another marker of muscular injury, was not different between the two groups (*P*-value: n.s.). Both groups showed increasing trends of these parameters postoperatively. The frequency of rescue analgesic use was not different between the two groups (*P*-value: n.s.) (Table 2).

**Table 2:** Comparison of clinical and laboratory outcomes

	Preoperative	Postoperative Day 1	Postoperative Day 2	Postoperative Day 4	Postoperative Day 7
CPK (mcg/L)					
Group E	N/A	140 ± 11	156 ± 35	97 ± 14	66 ± 6
Group C	N/A	105 ± 22	140 ± 17	81 ± 8	62 ± 5
*P*-value	N/A	n.s	n.s	n.s	n.s
LDH (IU/L)					
Group E	N/A	202 ± 57	211 ± 62	247 ± 58	277 ± 58
Group C	N/A	200 ± 40	208 ± 49	244 ± 55	271 ± 81
*P*-value	N/A	n.s	n.s	n.s	n.s
Hemoglobin (g/dL)					
Group E	11.9 ± 1.7	10.1 ± 1.5	9.5 ± 1.2	9.6 ± 1.1	9.7 ± 1.0
Group C	11.8 ± 1.8	9.9 ± 1.4	9.4 ± 1.2	9.4 ± 1.3	9.5 ± 1.0
*P*-value	n.s	n.s	n.s	n.s	n.s
Hematocrit (%)					
Group E	36.6 ± 5.1	30.8 ± 4.4	29.1 ± 3.5	29.7 ± 3.5	30.1 ± 3.0
Group C	35.3 ± 5.9	30.1 ± 4.0	28.7 ± 3.7	28.9 ± 3.9	29.2 ± 3.1
*P*-value	n.s	n.s	n.s	n.s	n.s
Rescue analgesic user (%)					
Group E	N/A	41.7	66.7	58.3	33.3
Group C	N/A	39.6	66.7	60.4	16.7
*P*-value	N/A	n.s	n.s	n.s	n.s

The data are expressed as the mean ± standard deviations

*Group E* elastic cuff group, *Group C* conventional cuff group, *CPK* creatine phosphokinase, *LDH* lactate dehydrogenase, *n.s* not significant, *N/A* not applicable

### Recommended tourniquet pressure, bloodlessness of surgical field, and surgical time

The RTPs for Group E and Group C were not significantly different (215 mmHg ±37 vs. 221 mmHg ±52, respectively, *P*-value: n.s.) The differences between RTP and the SBP for Groups E and C were 78 (±40) mmHg and 96 (±44) mmHg, respectively (*P* = 0.045). The bloodlessness of the surgical field was not different between the two groups (*P*-value: n.s.). In one case in Group E, and in two cases in Group C, tourniquet pressure had to be manually increased due to a poor surgical field (Table 3). Surgical time was not different between the two groups (51.4 min ±13.0 vs. 50.0 min ±12.3, respectively, *P*-value: n.s.).

**Table 3:** Comparison of intraoperative and postoperative outcomes

	Group E (*n* = 48)	Group C (*n* = 48)	*P*-value
RTP (mmHg)	215 ± 37	221 ± 52	n.s
RTP – SBP (mmHg)	78 ± 40	96 ± 44	0.04
Bloodlessness of surgical field (n, %)			n.s
Excellent	8 (16%)	6 (12%)	
Good	36 (74%)	41 (72%)	
Fair	4 (8%)	6 (12%)	
Poor	1 (2%)	2 (4%)	
Skin complication	0	0	N/A
DVT (n, %)	5 (10%)	12 (24%)	n.s
DVT of the thigh	0 (0%)	1 (2%)	n.s
DVT of the calf	5 (10%)	11 (22%)	n.s

The data are expressed as the mean ± standard deviations

*Group E* elastic cuff group, *Group C* conventional cuff group, *RTP* recommended tourniquet pressure, *SBP* systolic blood pressure, *DVT* deep vein thrombosis, *n.s* not significant, *N/A* not applicable

### Complications

None of the patients in either group experienced postoperative skin complications such as bullae of the thigh or wound infections.

On postoperative day 7, DVT in the lower extremity occurred in five patients (10.2%) in Group E; DVT in all these cases occurred in the calf. DVT also occurred in 12 patients (24.5%) in Group C; among these, DVT occurred in the thigh in one case (Table 3).

## Discussion

The most important finding of the present study is that a tourniquet system using an elastic cuff and LOP (compared with a tourniquet using a conventional cuff and LOP) reduced early postoperative thigh pain after TKA. The incidence of proximal DVT, although not significantly different, was lower with the use of an elastic cuff.

Thigh pain has been considered a minor complication after tourniquet use. However, thigh pain following TKA surgery has been one of the most common early postoperative complaints, and can contribute to patient dissatisfaction after TKA [1, 3, 15, 16]. In a prospective randomized controlled study which compared tourniquet use versus non-tourniquet use in TKA, results showed reduced thigh pain in the non-tourniquet group on postoperative day 4 [1]. This result corresponds well with the results of the present study, because thigh pain decreased significantly on postoperative days 4 and 7, facilitating the rehabilitation process of the patient. The elastic cuff, which reduces pinching, may account for the decreased thigh pain after surgery.

DVT after TKA is a serious complication. The incidence of DVT after TKA ranges from 40 to 84% [17]. Increased DVT with the use of a tourniquet is theoretically explained by increased venous stasis and endothelial damage. However, studies have reported that application of a tourniquet does not increase the risk of DVT [16, 18]. Proximal DVT has been associated with an increased risk of symptomatic pulmonary embolism [19], whereas DVT below the knee level has controversial associations with pulmonary embolism [20]. In the present study, the incidence of DVT was not significantly different between the two groups. However, there was one case of proximal DVT in the conventional tourniquet group which needed treatment, whereas no cases of proximal DVT were found in the elastic cuff group. Although not statistically significant, these results may prompt a further study investigating the effect of tourniquet material on the incidence of DVT.

Skin blisters of the thigh after tourniquet application during TKA can be a source of substantial morbidity postoperatively [21]. In a previous study, placing an elastic stockinet under the thigh resulted in significantly fewer skin complications. Elastic stockinet use has been known to produce fewer pinches and wrinkles [22]. In the present study, no skin complications were observed at the site of tourniquet application. The elastic cuff made of silicone rubber may have played a role in decreasing skin complications such as thigh pain, because less pinching of the skin is expected for this cuff type than that for a conventional cuff.

Studies comparing the raw material of the cuff with an aim to reduce the complications associated with tourniquet use have rarely been reported. Several studies have reported a silicone ring tourniquet as an alternative to the standard pneumatic tourniquet [23–25]. A recent randomized comparative study compared silicone ring tourniquets to conventional cuff tourniquets in TKA [15]. However, the above mentioned study only compared the serum lactate level, which was not significantly different to that observed with a pneumatic tourniquet. The above mentioned study also did not report results for other variables such as thigh pain, operative field quality, or for complications such as DVT. Silicone ring tourniquets have the advantage that the concentric force of the silicone ring reduces skin wrinkling; this potentially reduces soft tissue injury, similar to the elastic cuff in the present study. An elastic cuff which is composed of silicone is able to deliver the pneumatic force more efficiently than a conventional cuff, due to less pinching of the cuff.

There are some limitations to this study. First, the incidence of DVT is known to vary among different ethnic groups. Asians are reported to have a lower incidence of DVT than do people of other ethnicities [26]. The patients in the present study were all Asians. Therefore, the results for DVT cannot be generalized to all ethnicities. Second, bloodlessness of the surgical field was determined by the surgeon’s subjective opinion. Especially, the decision to raise the tourniquet pressure due to poor visualization was made with subjective judgement by the surgeon, who was not blinded to the treatment allocation. Third, analgesic usage was expressed as the percentage of patients using rescue analgesics, so the number of patients may have been inadequate for detecting significant differences between the two study groups.

## Conclusions

In conclusion, this prospective randomized clinical trial showed that the tourniquet system using an elastic cuff and LOP reduced early postoperative thigh pain after TKA compared to the tourniquet system using a conventional non-elastic cuff.

## Data Availability

Data and materials can be accessed through a request to the lead author.

## Abbreviations

DVT: Deep vein thrombosis
LOC: Limb occlusion pressure
TKA: Total knee arthroplasty
VAS: Visual analog scale
RTP: Recommended tourniquet pressure
CPK: Creatine phosphokinase
LDH: Lactate dehydrogenase
SBP: Systolic blood pressure
SDs: Standard deviations
ANOVA: Analysis of variance
n.s: Not significant
